# Editorial: Congenital anomalies: State of the art and the new paradigms for a precision public health approach

**DOI:** 10.3389/fped.2022.968923

**Published:** 2022-07-18

**Authors:** Babak Khoshnood, Gareth Baynam, Maria Loane, Anke Rissmann, Lorenzo Botto

**Affiliations:** ^1^INSERM U1153 Centre de Recherche Épidémiologie et Statistique, Paris, France; ^2^University of Western Australia, Perth, WA, Australia; ^3^Faculty of Life and Health Sciences, Ulster University, Northern Ireland, United Kingdom; ^4^Otto-von-Guericke University, Magdeburg, Germany; ^5^University of Utah Medical Center, Salt Lake City, UT, United States

**Keywords:** congenital anomalies (CAs), epidemiology, precision medicine, public health, outcomes

Congenital anomalies (CA) can be broadly defined as structural, anomalies, chromosomal anomalies, and syndromes occurring in the early stages of embryonic development that may be diagnosed prenatally or postnatally. They are a major cause of perinatal mortality, childhood morbidity and childhood disability. The scholarly activities concerned with CA comprise a complex set of scientific questions and public health challenges. A rich mix of disciplines as diverse as genetics, embryology, pathology, physiology, public health, epidemiology, biostatistics but also social sciences including sociology, psychology, medical ethics and anthropology can and do address important questions concerning fetuses and newborns with CA.

Our topic includes 16 manuscripts that address a limited but coherent set of topics as may be appropriate for a Research Topic on CA coordinated by our colleagues at Frontiers in Pediatrics. Our focus was, broadly speaking, public health issues with important contributions from population-based epidemiological studies, systematic reviews and meta-analyses. We also included interesting clinical case studies and crucial considerations on data quality in studies of CA. We were also fortunate to include a qualitative study on the important topic of parents' perspectives on newborns with CA. Finally, we included articles that included questions related to CA but were not exclusive to these anomalies. Even if they went beyond the field of CA, they also shared important overlapping features with the literature on CA.

Precision public health is a relatively recently used term with, forgive the pun, at times imprecise definitions and connotations. Our goal here was to be precise about the research questions tackled and the methods required to address them and their limitations. In general, as proposed by Khoury et al. precision public health refers to a population approach (i.e., the population is the unit of intervention rather than the individual) for providing the right “treatment” at the right time to the right people. Of course, from a population perspective, for conditions such as CA, “treatment” must be expanded to include the triple focus on causes (e.g., prevention), occurrence, and outcomes (including the evaluation of care and the societal impact on families and health care services).

An important and often quite difficult issue to address in studies of CA is the inherent, multi-dimensional heterogeneity of these anomalies. Indeed, CA represent a diverse set of pathologies in terms of their empirical attributes and epidemiology—wide variations in their total and live birth prevalence, known environmental risk factors and teratogenic effects of medications, surveillance methods, coding schemes, anatomy, pathophysiology, fetal vs. newborn physiology (notably in the case of congenital heart defects), embryology and organogenesis. There are multiple and myriad combinations of the modalities for medical and surgical management of the anomalies, substantial differences in the short- and longer- term health care and educational requirements of the different anomalies and varying degrees of progress in the prenatal screening, pre and post-natal management and the attendant trends in the short and long-term health and developmental outcomes of CA.

Even within groups of CA, perhaps most notably, for congenital heart defects, there are great heterogeneities with respect to the aspects noted above for CA in general. The article concerned with the trends in the prognosis of hypoplastic left heart syndrome (Best et al.) represents one “extreme” case of CHD as compared to the fortunately far more frequent minor ventricular septal defects that almost always resolve spontaneously with no health repercussions for the newborns with this particular type of CHD. There are also “intermediate” but certainly major subtypes of CHD, most notably Tetralogy of Fallot, Transposition of Great Arteries and Coarctation of Aorta. For the CHD in general and the subtypes mentioned above, substantial progress has been made for their prenatal diagnosis, postnatal screening (by pulse-oximetry in particular) and in their medical and surgical management. As a result, temporal trends clearly show improved outcomes for these anomalies even if long-term neuro-developmental and other health and education outcomes remain important questions that are being actively studied as part of long-term, prospective, population-based cohort studies.

With the exponentially increasing rate of accumulation of literature in general, and specifically in the field of CA, systematic reviews and meta-analyses, when appropriate, can make important contributions to the literature as represented by the systematic review of studies on the relation between CA and growth restriction at birth (Ghanchi et al.) in this Research Topic. Such reviews also have the added advantage of pointing out limitations in the currently available literature and can suggest fruitful venues for future research.

An important area of research reflects sociodemographic differences in relation to access to health care services such as prenatal screening (Santoro et al.). Other important areas of public health and epidemiological research relate to “Prevention” of CAs such as introducing folic acid fortification in Europe. The effect of not introducing mandatory fortification on the prevalence of neural tube defects is highlighted in the European study by Morris et al.

Ebert et al. demonstrated the value of health care data to determine the prevalence of rare diseases such as bladder exstrophy and epispadias. However, for research related to congenital heart defects, population-based registry data were obtained to assess the risk of growth restriction at birth (Ghanchi et al.) and to demonstrate the improved prognosis in children with hypoplastic left heart (Best et al.).

In conclusion, overall, CA represent relatively frequent events (3% of all births) with important, broadly speaking public health issues. As evident in this sample of studies, there is great heterogeneity within CA and most CA are rare diseases however (prevalence <1 per 2,500 births) and hence their study represents important conceptual and empirical challenges. In this context, it is particularly important for studies to be as precise as possible about the scientific question(s) at hand, appropriate study design and analysis and the limits of each study. It is also essential that we understand whether and to what extent results a given study on a specific type or subgroup of CA may or may not be applicable to other CA. Notwithstanding these caveats, the literature on CA continues to grow and diversify in ways that move the field forward in both its scientific and public health endeavors.

## Author contributions

A first draft of the Editorial was prepared by BK and LB. All authors contributed to revision of the text and have read and approved the final version of the Editorial.

## Conflict of interest

The authors declare that the research was conducted in the absence of any commercial or financial relationships that could be construed as a potential conflict of interest.

## Publisher's note

All claims expressed in this article are solely those of the authors and do not necessarily represent those of their affiliated organizations, or those of the publisher, the editors and the reviewers. Any product that may be evaluated in this article, or claim that may be made by its manufacturer, is not guaranteed or endorsed by the publisher.

